# Applying the Ensemble and Metaheuristic Algorithm to Predict the Flexural Characteristics of Ice

**DOI:** 10.3390/ma19020333

**Published:** 2026-01-14

**Authors:** Chengxi Lu, Xiangyu Han

**Affiliations:** 1 School of Information and Control Engineering, Southwest University of Science and Technology, Mianyang 621000, China; 2 School of Civil Engineering and Architecture, Southwest University of Science and Technology, Mianyang 621000, China

**Keywords:** flexural properties, ice, ensemble algorithms, firefly metaheuristic algorithm, feature importance

## Abstract

The stability of ice structures in cold regions and polar environments has been increasingly challenged by global warming and climate change, making the accurate estimation of ice flexural properties essential. However, the flexural failure process of ice is highly complex, and the calculated flexural properties are influenced by multiple factors. Hence, several data-driven artificial intelligence models were developed to predict flexural strength, using classification and regression tree (CART), AdaBoost, and Random Forest methods, while the Elitist Ant System (EAS) was applied to optimize model parameters. The EAS procedure converged rapidly within ten iterations and effectively enhanced overall model performance. Compared with the single CART model, ensemble approaches exhibited higher prediction accuracy and better generalization, with AdaBoost achieving the best performance (R^2^ = 0.736). Feature-importance analysis indicated that the testing method and specimen geometry had the greatest influence on the results, highlighting the importance of careful control of experimental conditions. The proposed ensemble–metaheuristic framework provides an efficient tool for predicting the mechanical behavior of ice and offers useful support for stability assessments of ice structures under changing climatic conditions.

## 1. Introduction

Over recent decades, global warming has drawn attention due to impacts like ice melt and biodiversity loss, mainly driven by fossil-fuel use in transport, industry, and energy systems [1,2,3]. In cold regions and polar areas that are mostly covered with ice, climate change’s impact on the stability of ice structures cannot be ignored [4,5]. Due to their numerous defects, bubbles, and voids, ice structures often fracture under very light flexural loads [6,7,8,9]. Hence, the flexural properties of ice are critical factors in determining the bearing capacity of an ice structure.

Many studies have investigated the flexural properties of ice, including indoor experiments, outdoor tests, theoretical research and numerical simulations [10]. Gow et al. used cantilever and simply supported testing methods to study the effect of structure and temperature on the flexural properties of laboratory ice sheets [11]. Dempsey et al. conducted a set of lab-to-structural-scale fracture tests on freshwater lake ice to study the effects of size on tensile strength and fracture, with the results showing a remarkable correlation between the microstructure of ice and the failure processes of the samples [12]. Timco et al. compiled the results of 2495 flexural experiments on freshwater and sea ice and established a correlation between flexural strength and brine volume [13]. Ji et al. simulated flexural tests of sea ice using the discrete element method and studied how crystalloids influence the macro-mechanical properties of sea ice [14].

Although the above research has made significant progress, the measurement and prediction of the flexural strength of ice remains challenging due to the complexity of its microstructure. Laboratory experiments are limited by the small size of specimens, which makes it difficult to account for the heterogeneity and polycrystalline nature of ice. As a result, the measured flexural strength cannot reliably be extrapolated to samples of different sizes. Field tests, while they avoid such size effects and better reflect the actual mechanical behavior of ice by using larger specimens, suffer from the inability to effectively control environmental conditions. Other approaches also face limitations; empirical formulas are generally valid only for specific cases, and numerical simulations often encounter difficulties in parameter calibration.

Artificial intelligence (AI), inspired by human cognitive and reasoning processes, has emerged as a powerful paradigm for data-driven prediction and decision-making [15]. It has demonstrated remarkable potential across diverse engineering domains, including civil, mechanical, and transportation engineering [16,17,18]. By learning the intrinsic relationships between input and output variables, AI models can perform reliable predictions without requiring explicit formulation of the underlying physical mechanisms. To further enhance prediction accuracy and model stability, ensemble learning algorithms such as AdaBoost and random forest combine multiple weak learners into a single strong predictive framework, effectively mitigating overfitting and improving generalization [19,20,21]. In parallel, metaheuristic optimization techniques have been increasingly employed to search for optimal hyperparameter configurations, further refining the predictive efficiency and robustness of AI models [22,23]. Despite the above potential, the use of data-driven predictive modeling to estimate the flexural behavior of ice remains limited in current research. Existing studies are generally constrained by small datasets, limited interpretability, and an absence of integrated treatments of geometric, microstructural, and experimental variables within a unified modeling framework.

In this study, artificial intelligence (AI) techniques are employed to develop predictive models for estimating the flexural strength of ice, with the primary contribution lying in the establishment of a systematic data-processing and machine-learning methodology rather than in redefining known physical relationships. Unlike conventional studies that rely mainly on analytical beam theory or empirically derived mechanical models, this work emphasizes data sorting, feature screening, and the comparative evaluation of multiple machine-learning algorithms optimized using the Elitist Ant System (EAS). This approach enables the identification of dominant influencing factors and provides a robust framework for handling complex experimental datasets.

The structure of the paper is organized as follows. Section 2 introduces the theoretical background of the three selected machine-learning algorithms and the EAS optimization approach. Section 3 and Section 4 describe the dataset construction and data preprocessing procedures adopted for model training. Section 5 presents the model development process. Section 6 compares the predictive performance of the trained models and analyzes the relative importance of key features affecting the flexural behavior of ice. Finally, Section 7 summarizes the main conclusions, scientific contributions, and implications for future research.

## 2. Machine Learning Algorithms

To select appropriate machine learning algorithms for this study, the specific characteristics of the ice test dataset should be carefully considered. The dataset contains a mixture of numerical and categorical variables, exhibits moderate size, and includes experimental variability inherent to laboratory ice testing. Tree-based methods were therefore chosen because they handle heterogeneous input types without strict data-normalization requirements, maintain stable performance on datasets of limited size, and provide transparent feature importance measures that support physical interpretation of influential factors.

### 2.1. Classification and Regression Tree

Classification and regression tree (CART) is a fundamental type of decision tree learning algorithm widely used in both classification and regression tasks [24,25,26]. CART constructs a binary decision tree by repeatedly partitioning the dataset according to feature values that best separate the data. At each node, a feature and a corresponding split point are chosen so that the resulting two subsets show the smallest possible variation in the target variable, leading to progressively more uniform data groups as the tree grows. The splitting continues until a predefined stopping criterion is met, such as the minimum number of samples in a node or a maximum tree depth. For classification problems, the final prediction is the class label that appears most frequently in the leaf node. For regression problems, the output is the average of the target values in the leaf node. CART is known for its interpretability, flexibility, and ability to handle both numerical and categorical data, although it is prone to overfitting when used as a standalone model.

### 2.2. Bagging Ensemble Algorithm

The bagging ensemble algorithm is a kind of ensemble learning method which combines several weak learners into a strong learner. The training data are randomly selected from the dataset with replacement, which means the same instance may be selected multiple times. Then, the weak learners are trained independently and in parallel with each other. Various weak models are combined together to construct the strong learner with high prediction accuracy and generalization ability.

**Figure 1:**
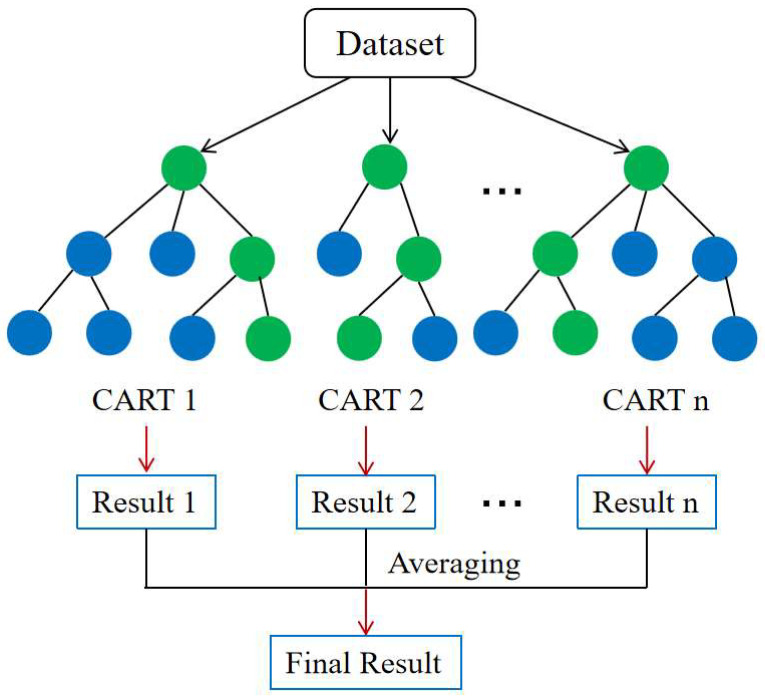
Illustration of random forest algorithm.

Random forest (RF) is one of the most famous and commonly used bagging methods [27,28,29]. As shown in Figure 1, a large number of individual decision trees are created by using bagging and feature randomness. Each decision tree is trained on a randomly sampled subset of the dataset, and each tree produces its own prediction. For regression tasks, the final output is obtained by averaging the predictions from all trees, while for classification tasks, the class receiving the majority of votes is selected. By aggregating the results of multiple weak learners, this approach effectively reduces model variance and improves overall prediction accuracy.

### 2.3. Boosting Ensemble Algorithm

Boosting is another widely used ensemble learning technique. For the boosting algorithm, the weak learners are trained sequentially, and the subsequent prediction tries to compensate for the weaknesses of its predecessor [30]. After several iterations, a strong learner with low bias is constructed. There are two issues to be solved for each boosting learning methods: adjusting the weight or distribution of training data in each iteration, and integrating the weak learners.

**Figure 2:**
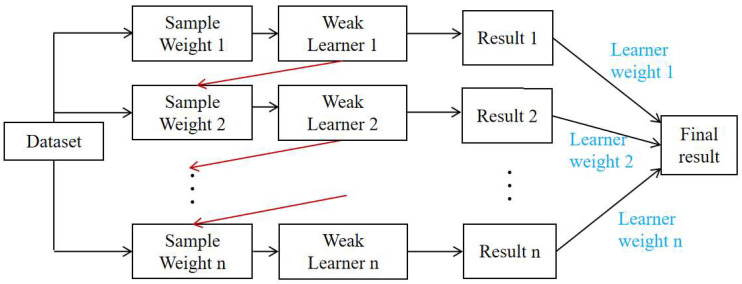
Illustration of the random Adaboost algorithm.

AdaBoost is an iterative learning approach in which greater emphasis is placed on samples that are difficult to predict, as shown in Figure 2. After each iteration, samples that were previously mispredicted are assigned higher importance, while correctly predicted samples receive reduced weights, directing subsequent learners toward the more challenging cases. This progressive reweighting allows the model to correct earlier mistakes and refine its overall performance. After a sequence of learners has been established, their outputs are combined through a weighted voting scheme, in which learners with lower prediction errors exert a stronger influence on the final prediction. As a result, the method gradually improves prediction accuracy while maintaining good generalization capability, particularly when handling complex and heterogeneous datasets.

### 2.4. Elitist Ant System Algorithm (EAS)

**Figure 3:**
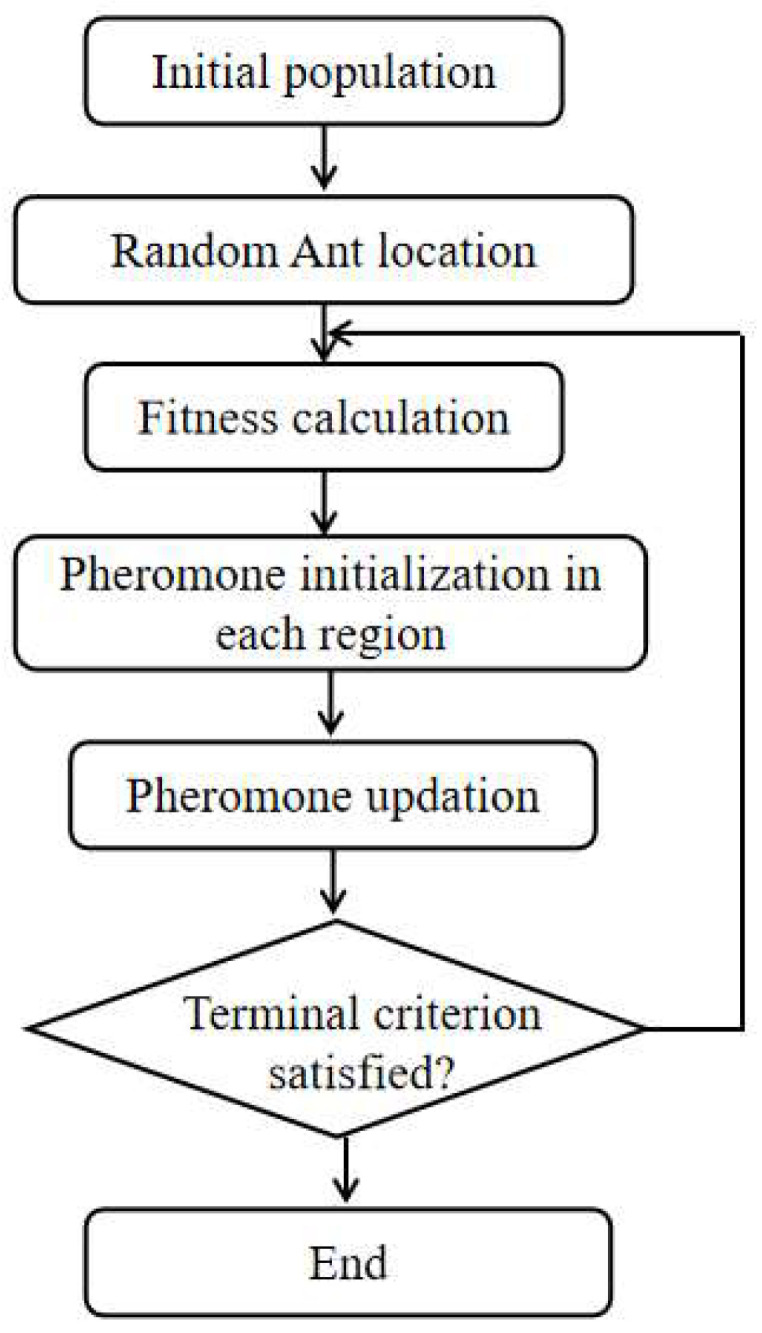
Schematic diagram of the ant colony optimization algorithm.

The ant colony optimization (ACO) is a population-based metaheuristic which is inspired by the instinctive behavior of ants [31,32]. Figure 3 describes the essential procedure of ACO. During foraging, ants will deposit a chemical substance called pheromones. The ant colony is sensitive to the intensity of pheromones, and follows paths with higher amounts of pheromone. The shorter paths are always accompanied by more intense pheromones, and so these paths will be chosen by more ants. Thus, positive feedback is established, and the optimum path from nest to food will be found.

The elitist ant system (EAS) is an improved version of ACO which enhances the converging rate by reinforcing the pheromone intensity of elitist ants (those on the optimum path). When the pheromone intensity of all the paths is first initialized, every path is given the same amount of pheromone, which means every path is equally attractive to the ant colony. When the algorithm iterates to round *t*, the ant *k* at location *i* will face the choice of the next location *j.* The state transition follows the rules in Equation (1) [31].

(1) $$p_{ij}^{k}=\left\{\begin{array}{c} \frac{\tau_{ij}^{\alpha }(t)\eta_{ij}^{\beta }(t)}{\sum_{k\in \;allowed_{k}}\tau_{ij}^{\alpha }(t)\eta_{ij}^{\beta }(t)},\;j\in \;allowed_{k}\;\\ 0,\;\mathrm{otherwise} \end{array}\right.$$

where $p_{ij}^{k}$ represents the possibility of ant *k* at location *i* choosing location *j*; $\tau_{ij}(t)$ represents the pheromone intensity of path *ij*; α reflects the impact of pheromone on the colony’s path selection; $\eta_{ij}(t)$ is the heuristic function defined by the inverse of distance between location *i* and *j*; β is the expected heuristic factor; $allowed_{k}$ represents the paths that ant *k* never passes.

After the ant colony finishes round *t*, all the paths are compared to select the best route *T^gb^* with the least distance *L^gb^*, and the corresponding ant is elected as the elitist. Then, the pheromone intensity of round (t + 1) is determined by three parts: the previous pheromone $\tau_{ij}(t)$, the pheromone deposited by ant k during the *ij* tour $\Delta \tau_{ij}^{k}(t)$, and the award for the elitist ant $\Delta \tau_{ij}^{gb}(t)$. The detailed updating rule can be seen in Equation (2) [31]:

(2) $$\tau_{ij}(t+1)=(1-\rho )\tau_{ij}(t)+\sum_{k=1}^{m}\Delta \tau_{ij}^{k}(t)+\Delta \tau_{ij}^{gb}(t)$$

where ρ is the evaporation coefficient in the range of 0 to 1.

$\Delta \tau_{ij}^{k}(t)$ can be calculated as follows:

(3) $$\Delta \tau_{ij}^{k}(t)=\left\{\begin{array}{c} 1/L^{k}(t),\;(i,j)\in T^{k}\\ 0,\;\mathrm{otherwise}\; \end{array}\right.$$

where $T^{k}$ represents route that ant *k* travels from *i* to *j*, and $L^{k}(t)$ is the distance of $T^{k}$.

$\Delta \tau_{ij}^{gb}(t)$ can be calculated as follows:

(4) $$\Delta \tau_{ij}^{gb}(t)=\left\{\begin{array}{c} e/L^{gb}(t),\;(i,j)\in T^{*}\\ 0,\;\mathrm{otherwise}\; \end{array}\right.$$

where *e* is encouraging coefficient.

After the ant *k* finishes the journey from location *i* to *j*, communication between ant k and other ants is established by updating the pheromone intensity of the most recent path of ant k. The local state updating activity follows the rule in Equation (2):

Where ρ is the evaporation coefficient in the range of 0 to 1, and $\Delta \tau_{ij}^{k}(t)$ represents the pheromone deposited by ant k during the *ij* tour.

As the travel of the ant colony is determined by the state transition rule and state update rule, and led by the enlist ant, the global optimum will be found after several iterations.

## 3. Data Collection

The dataset employed for developing and validating the predictive models was collected from experimental studies conducted by the U.S. Army Cold Regions Research and Engineering Laboratory (CRREL) [11]. These experiments systematically investigated the flexural behavior of ice and the factors influencing its mechanical performance. In total, 730 small-scale ice beams were tested under diverse experimental conditions involving variations in beam geometry, temperature, testing methodology, tensile surface orientation, and specimen preparation. The statistical distribution of the experimental parameters and corresponding test results is summarized in Table 1. Four data points were excluded from analysis due to incomplete or missing information (e.g., missing test method specification, failure loads).

**Figure 4:**
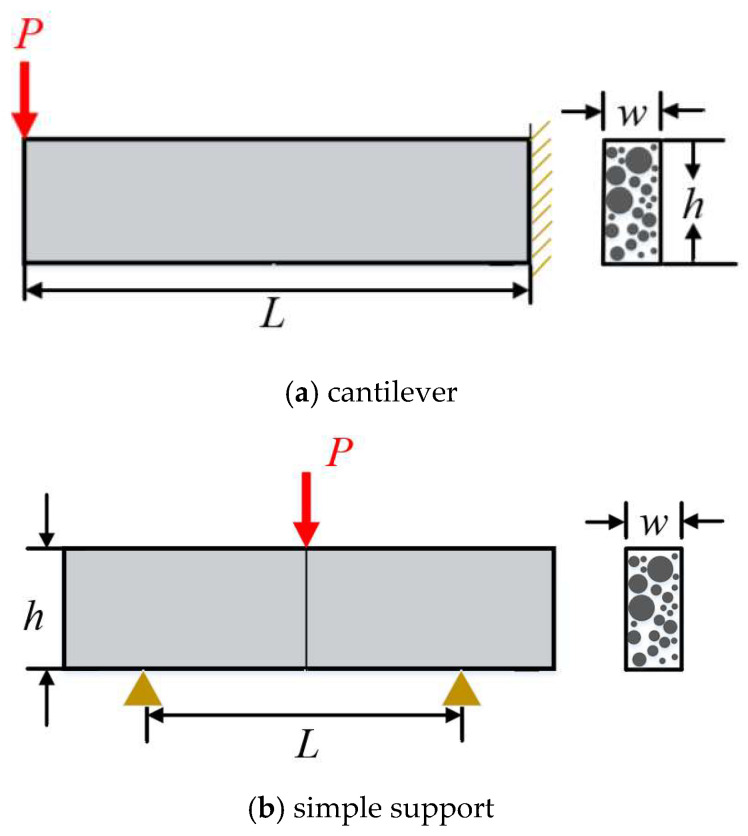
Schematic illustration of loading conditions for (**a**) cantilever and (**b**) simple support.

Additional details for the variables summarized in Table 1 are provided as follows. Temperature measurements correspond to the recorded temperature at the top surface of each specimen at the time of testing. Four testing methods were included in the dataset: cantilever, modified cantilever, parallel simple support, and isothermal simple support. The modified cantilever configuration incorporated stress-relief holes near the fixed end to mitigate stress concentrations during loading. Specifically, the normally sharp corners at the roots of conventional cantilever beams, originally formed by parallel saw cuts, were modified by drilling circular holes with a diameter of 20 cm. This geometric modification resulted in a smoother cross-sectional profile and promoted a more uniform stress distribution during bending. The isothermal simple-support method represents a variant of the parallel simple-support configuration in which temperature is maintained uniformly along the entire beam length. The typical loading conditions and support/boundary constraints for the cantilever and simple support are illustrated in Figure 4. The tensile surface was classified as either the top or bottom surface, depending on which side experienced tension during bending. In addition, the seeded samples refer to specimens prepared by introducing frozen droplets onto the mold surface prior to freezing, thereby promoting controlled nucleation during ice formation. A more detailed description of the experimental conditions and testing procedures is available in Ref. [11].

The flexural strength of ice specimens could be calculated as follows,

(5) $$S_{f}=\frac{6PL}{wh^{2}}\;\;(\mathrm{cantilever})$$

(6) $$S_{f}=\frac{3PL}{2wh^{2}}\;\;(\mathrm{simple}\;\mathrm{support})$$

where *P* is the failure load, *L* is the length of the beam, *w* and *h* are the width and thickness, respectively.

## 4. Data Preprocessing and Preparation

Data preprocessing is indispensable for machine learning. As shown in Table 1, the variables include both numeric and nominal values. The numeric data can be directly fed into the predictive models. However, nominal data, which cannot be ordered or measured, cannot be received by the predictive models. Hence, the nominal variables (test method, tensile surface, and specimen preparation) were transformed into numerical form prior to model training. Label encoding was adopted to assign each categorical level a unique integer, ensuring compatibility with the tree-based predictive models while preserving the distinct identities of the categorical attributes. Accordingly, different experimental conditions are regarded as input variables, and the test results are set as output variables.

**Figure 5:**
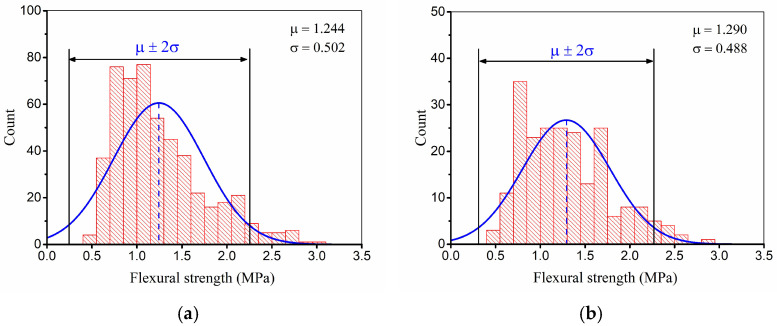
Distribution of datasets: (**a**) training; (**b**) testing.

Data partitioning is another crucial step in data preparation, it divides the dataset into different subsets to be used for different purposes in machine learning. The training data are used to train the model; the model learns from the patterns in the training set and adjusts its parameters to optimize its performance. The testing data are used to estimate the performance of the model on unseen data after the model is established. To maintain data representativeness, the dataset was randomly shuffled prior to partitioning. Subsequently, 70% of the samples (509 records) were allocated for model training, and the remaining 30% (208 records) were reserved for testing. To verify the statistical consistency between the two subsets, a normality analysis was performed, as illustrated in Figure 5. The training data exhibited a mean flexural strength of μ = 1.244 MPa and a standard deviation of σ = 0.502 MPa, whereas the testing data showed μ = 1.290 MPa and σ = 0.488 MPa. Despite minor differences, both subsets follow comparable distribution patterns, confirming that the partitioned data are sufficiently representative for reliable model development and validation.

## 5. Model Construction Procedure

### 5.1. Cross Validation Methods

Given a specific training dataset and learning algorithm, varying the parameter settings can result in multiple models. Consequently, identifying the optimal set of parameters becomes a critical step in model development. Cross-validation is commonly employed to assess a model’s generalization capability and to guide the selection of the most suitable parameter combination [33].

**Figure 6:**
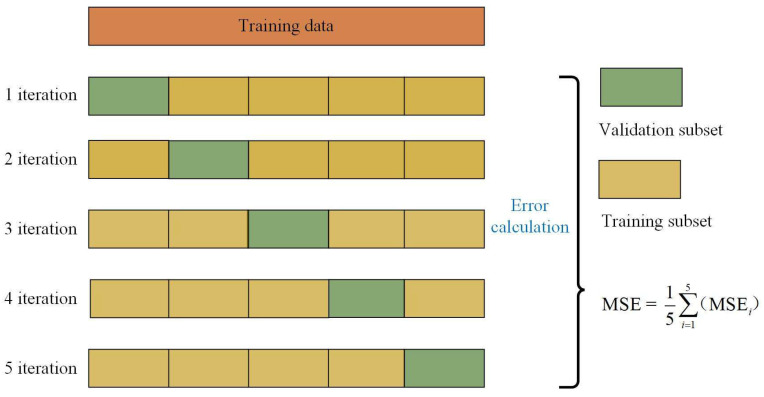
Schematic diagram of 5-fold cross-validation during training procedure.

To make the most use of the data, K-fold cross-validation is applied here. As shown in Figure 6, the data set is roughly split into K folds of equal size. K-1 of the folds are used to train the model, and the remaining fold is used for evaluation. In this way, the training and evaluating process are repeated K times, each time with a different validation set. After K iterations, the performance of the model is typically summarized by taking the average of the performance metrics computed in each fold. In this study, a 5-fold cross-validation approach was adopted to assess model performance due to the good balance between bias and variance in performance estimation for datasets of this size. The mean squared error (MSE) was selected as the evaluation metric, and its average value over the five iterations was used to quantify the predictive accuracy, as expressed in Equation (7).

(7) $$MSE=\frac{1}{N}\sum_{n=1}^{N}{\left(y_{n}-{\hat{y}}_{n}\right)}^{2}$$

In Equation (7), $y_{n}$ denotes the experimental output, ${\hat{y}}_{n}$ denotes the predicted output, and *N* is the number of samples. The value of *MSE_i_* is computed for each i-th fold during the cross-validation process, and the average value (*MSE_av_*_g_) across all folds is subsequently used to evaluate the overall predictive performance of the model.

### 5.2. Hyper-Parameter Tuning

As discussed above, the performance of models can be initially evaluated with cross validation and average mean squared error (*MSE_avg_*). Hence, the Elitist Ant System algorithm (EAS) can be utilized to find the optimum hyper-parameters by taking the *MSE_avg_* as the objective function. After tracing the *MSE_avg_* value in each generation, the optimum hyper-parameters can be easily determined when *MSE_avg_* reaches the lowest value.

**Figure 7:**
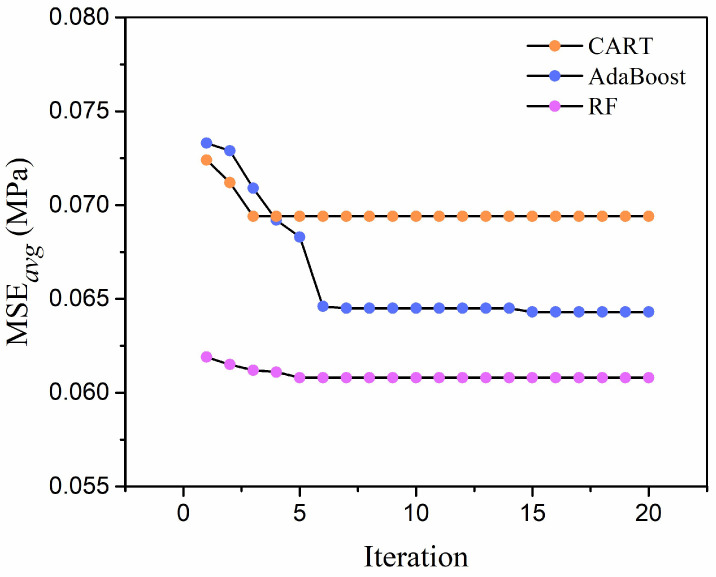
Evolution of *MSE_avg_* value in three models.

In addition to the RF and Adaboost algorithms, the CART algorithm is selected to construct the predictive models for comparison. With the help of training data, the *MSE_avg_* values of three predictive models within 20 iterations are collected. The evolution trends are also presented, as shown in Figure 7. It can be found that three models all converge to a stable state in 8 iterations, and the CART models converge to the optimal solution in the shortest time. The RF and Adaboost models took longer to converge to their optimal hyper-parameters, but ultimately achieved a lower *MSE_avg_* than the CART model. This is likely due to the fact that RF and Adaboost are ensemble models composed of multiple decision trees, and thus have a greater number of hyper-parameters to optimize. The EAS is proven to be an effective approach for hyperparameter tuning in machine learning.

The optimal hyper-parameters determined for the three predictive models are summarized as follows:

(1) CART: max_depth = 7, min_samples_split = 5, min_samples_leaf = 3.

(2) AdaBoost: max_depth = 8, min_samples_split = 7, min_samples_leaf = 4, n_estimators = 52.

(3) RF: max_depth = 15, min_samples_split = 9, min_samples_leaf = 7, n_estimators = 145.

## 6. Results and Discussion

### 6.1. Predictive Model Performance

Once the optimal hyper-parameters have been identified, the CART, random forest (RF), and Adaboost prediction models are also determined. Then, these fully trained models are evaluated with the testing dataset. The predictive models were fed with 218 input variables from the testing dataset, and the predictive results were outputted to a file. The predictive performance of these models can be quantitatively evaluated by comparing the real targets and output variables. Here, the coefficient of determination (R^2^) is selected to measure how well the predictive models predict the outcome, as shown in Equation (8).

(8) $$R^{2}=1-\frac{\sum_{n=1}^{N}{\left(y_{n}-{\hat{y}}_{n}\right)}^{2}}{\sum_{n=1}^{N}{\left(y_{n}-{\bar{y}}_{n}\right)}^{2}}$$

where $y_{n}$ denotes the real output variable, ${\hat{y}}_{n}$ denotes the predictive output variable, ${\bar{y}}_{n}$ denotes the average value of output variables in the experiment, and *N* is the data size.

**Figure 8:**
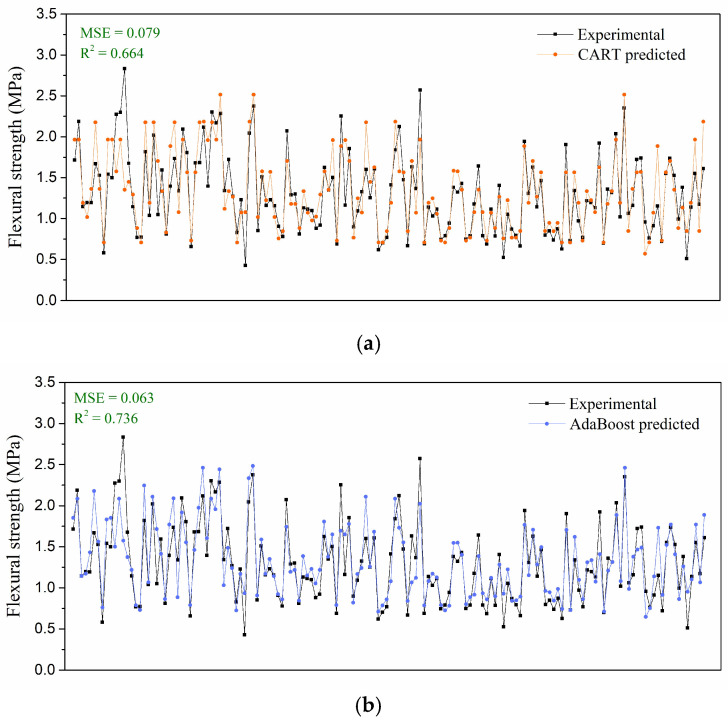

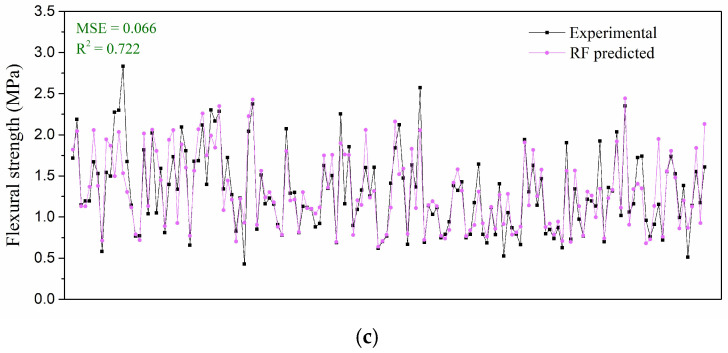
Comparison between experimental results and predictive results: (**a**) CART model; (**b**) AdaBoost models; (**c**) RF models.

Figure 8 illustrates the comparison between the experimental and predicted values obtained from different models, along with their corresponding *R^2^* coefficients. After hyperparameter optimization using the EAS algorithm, all three models demonstrated satisfactory predictive capability for the flexural strength of ice. Among these models, the AdaBoost model achieved the highest accuracy, with *MSE* = 0.063 and *R*^2^ = 0.736, whereas the CART model exhibited the weakest performance (*MSE* = 0.079, *R*^2^ = 0.664). Compared with its training behavior, the CART model showed a noticeable decline in prediction accuracy, while two ensemble methods maintained better generalization performance due to their boosting and bagging mechanisms.

### 6.2. The Importance of Different Influencing Variables

To better understand the dataset and the underlying predictive mechanisms, the relative importance of each input variable was analyzed after the model was built. As outlined in Section 5, the AdaBoost model, optimized using the EAS algorithm, showed the best predictive performance in both the training and testing stages. Hence, this model was used to assess how each feature contributes to the prediction of ice flexural strength.

**Figure 9:**
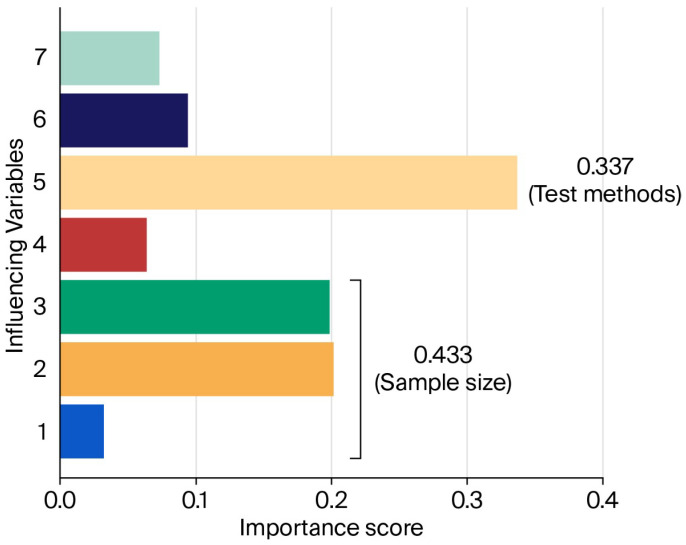
Relative importance of various influencing variables. (1: Length; 2: Width; 3: Thickness; 4: Temperature; 5: Test methods; 6: Tension surface; 7: Sample formation).

In this study, seven factors were selected as input variables: length, width, thickness, temperature, testing method, tension surface, and sample formation. As illustrated in Figure 9, these variables influence the performance of the AdaBoost prediction model to varying degrees. Specifically, length, width, and thickness were combined into a single variable termed sample size. The results show that sample size and testing method have the greatest impact on the model, with importance scores of 0.433 and 0.337, respectively.

The size of the sample has the strongest influence on predictive model, suggesting that the flexural strength of ice is heavily dependent on specimen dimensions. According to [11], the flexural strength of ice is generally calculated using elastic beam theory, which presumes that ice is a homogeneous and isotropic material.

**Figure 10:**
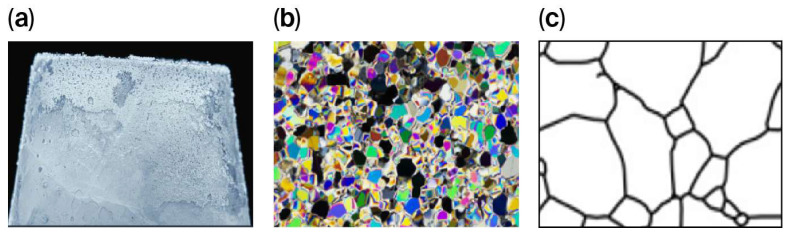
Multi-scale ice specimens at (**a**) meter, (**b**) centimeter, and (**c**) millimeter levels.

However, in laboratory experiments, the complex microstructure of ice often leads to test specimens that are too small to fully satisfy the assumptions of continuum theory. As a result, the measured flexural properties are inevitably influenced by the dimensions of the sample, as illustrated in Figure 10. The flexural fracture behavior of ice clearly depends on scale and is controlled by its internal structure. In large specimens on the meter scale, macrocracks are well developed, and the overall fracture process follows the principles of continuum mechanics. In contrast, centimeter-scale specimens are dominated by microcrack propagation, showing a noticeable size effect. When the specimen size decreases to the millimeter scale, grain boundaries and intrinsic defects impose strong restrictions, producing extremely brittle and discontinuous fracture patterns. Hence, sample size plays an essential role and should be treated as a key parameter when developing models to predict the flexural strength of ice. These observations are consistent with the findings reported in previous experimental and theoretical studies, which also identified pronounced size effects in the flexural and fracture behavior of ice and other quasi-brittle materials [34,35]. Compared with these studies, the present results further highlight that incorporating specimen size as an explicit input parameter in data-driven models can effectively capture scale-dependent fracture mechanisms and improve predictive accuracy across different length scales.

The test method is a critical factor influencing the measured flexural properties of ice. In this study, four bending methods are employed, including the cantilever, modified cantilever, parallel simple support and isothermal simple support methods. Under cantilever loading, stress tends to concentrate near the fixed end of the specimen, which often results in relatively lower measured strength values. To mitigate this effect, the modified cantilever introduces stress-relief holes at the beam root, thereby reducing stress concentration and improving the reliability of the measured results. In contrast, stress concentration is generally less pronounced in both simple support configurations. Furthermore, the isothermal simple support method extends the parallel simple support test by minimizing temperature gradients across the specimen thickness, leading to flexural properties that are more representative of the intrinsic mechanical behavior of ice. It is also noted that the stress states generated in these testing methods are not merely laboratory-specific conditions, but are commonly encountered in practical and natural scenarios of ice flexural failure. Therefore, the stress state should be explicitly considered when predicting ice flexural failure.

The other three input variables are temperature, tension surface, and sample formation, with importance scores of 0.063, 0.094, and 0.073, respectively. Among them, temperature contributed the least to the AdaBoost model’s prediction. This outcome may be attributed to two main factors. First, in order to replicate the formation of natural ice, a temperature gradient typically exists through the sample thickness, while only the temperature measured at the upper surface is recorded as the test temperature, leading to incomplete representation. Second, the experimental setup included only a limited number of temperature levels (−1 °C, −3 °C, −5 °C, −10 °C and −19 °C), which restricts variability in this feature and consequently reduces its influence on the model’s predictive performance.

## 7. Conclusions

This study developed a machine-learning framework for estimating the flexural strength of ice using tree-based predictive models combined with a metaheuristic hyperparameter optimization strategy. Beyond reporting predictive accuracy, the work demonstrates how experimental configuration, specimen characteristics, and testing methodology can be explicitly incorporated into data-driven models, providing complementary insights to conventional analytical approaches.

The main conclusions can be summarized as follows:

(1) Hyperparameter selection was shown to significantly influence model behavior. The Elitist Ant System (EAS) efficiently identified stable near-optimal parameter configurations within a limited number of iterations, highlighting its suitability for optimizing tree-based models with complex response surfaces.

(2) Ensemble learning methods (AdaBoost and Random Forest) consistently outperformed the single CART model in terms of prediction accuracy and generalization capability. Among the tested approaches, AdaBoost exhibited the most robust performance due to its sequential aggregation of weak learners and improved resistance to overfitting.

(3) Feature-importance analysis revealed that specimen geometry and testing method were the dominant influencing factors. While these dependencies are consistent with known physical principles, the results demonstrate that the proposed methodology can effectively capture and quantify their relative contributions within heterogeneous experimental datasets.

Overall, the primary contribution of this study lies in the methodological framework for data sorting, feature integration, and comparative machine-learning evaluation, rather than in redefining known mechanical laws. The proposed approach provides a transferable tool for analyzing complex material testing data, and it may be extended in future research to larger datasets, additional environmental variables, or hybrid physics-informed machine-learning models.

It should be noted that the scope of the dataset is limited by the availability of experimental data in the literature, and that the present study does not aim to provide a universal or exhaustive model covering all possible combinations of influencing factors. For future research, expanding the dataset to enhance representativeness and balance feature distribution is recommended. Moreover, integrating nonlinear elasticity and fracture mechanics could help refine both the accuracy and interpretability of the predictive models.

## Figures and Tables

**Table 1:** Variables of Ice Flexural Experiments.

Variables	Items	Data Type	Minimum	Maximum	Average	Standard Deviation
Conditions	Length (mm)	Numeric	71.10	120.00	101.05	8.32
	Width (mm)	Numeric	8.20	20.60	11.20	1.24
	Thickness (mm)	Numeric	6.40	18.90	10.22	1.49
	Temperature (°C)	Numeric	−19.00	−1.00	−7.29	6.11
	Test methods	Nominal	Cantilever, Modified cantilever, Parallel simple support, Isothermal simple support			
	Tension surface	Nominal	Top in tension, Bottom in tension			
	Sample formation	Nominal	Seeded, Unseeded			
Results	Flexural strength (MPa)	Numeric	0.35	3.07	1.26	0.50

## Data Availability

The data presented in this study are available on request from the corresponding author due to privacy.
